# Isolation and characterization of a genotype 4 Hepatitis E virus strain from an infant in China

**DOI:** 10.1186/1743-422X-6-24

**Published:** 2009-02-16

**Authors:** Wen Zhang, Shixing Yang, Quan Shen, Junfeng Liu, Tongling Shan, Fen Huang, Huibo Ning, Yanjun Kang, Zhibiao Yang, Li Cui, Jianguo Zhu, Xiuguo Hua

**Affiliations:** 1 Zoonosis and Comparative Medicine Group, School of Agriculture and Biology, Shanghai JiaoTong University, 800 Dongchuan Road, Shanghai, PR China

## Abstract

In the present study, a genotype 4 HEV strain was identified in the fecal specimen from a seven months old infant with no symptom of hepatitis in Shanghai Children's hospital. The full capsid protein gene (ORF2) sequence of this strain was determined by RT-PCR method. Sequence analysis based on the full ORF2 sequence indicated that this HEV strain shared the highest sequence identity (97.6%) with another human HEV strain isolated from a Japanese patient who was infected by genotype 4 HEV during traveling in Shanghai. Phylogenetic analysis showed that this genotype 4 HEV was phylogenetically far from the genotype 4 HEV strain that was commonly prevalent in Shanghai swine group, suggesting that this strain may not come from swine group and not involved in zoonotic transmission in this area.

## Findings

Hepatitis E virus (HEV), a member of the genus Hepevirus, is a non-enveloped virus with a positive-stranded RNA genome approximately 7.2 kb in length [1]. It is a major cause of acute epidemic and sporadic viral hepatitis in several developing countries. The infection is believed to be transmitted by the fecal-oral route, usually through contaminated drinking water. HEV isolates were divided into four distinct genotypes according to sequence and phylogenetic analyses. Genotype 1 was previously believed to only infect humans, but reportedly detected from a pig in Cambodia recently [2]. Genotype 2 has only been identified in humans in Mexico and Africa (Nigeria, Chad). Genotype 3 is prevalent in swine herds and humans all over the world. Genotype 4 HEV was first detected in humans in 1993 [3] and is mainly distributed in China, Japan, India, Indonesia, and Vietnam. Genotype 4 HEV has a wide host range, being prevalent in humans, swine, and some other animals. These four types of virus are thought to comprise a single serotype [4]. Hepatitis E was first recognized in China after a large epidemic in the south part of Xinjiang Uighur autonomous region [5]. Since 2000, genotype 4 HEV has become the dominant cause of hepatitis E disease in China [6].

Hepatitis E is usually self-limiting and its clinical illness is usually mild, though fulminant hepatic failure occurs in some cases. The disease is characterized by a high attack rate among young adults and particularly severe illness among pregnant women [5]. To our knowledge, no report so far indicates that infants have been infected by HEV in China and this is the first report that HEV infected the infant that was younger than one year old.

From Feb to Aug, 2008, 236 fecal samples were collected from 236 infants younger than 2 year old in Shanghai Children's Hospital, these infants were sent to the hospital because they had abnormal feces appearance for several days. All the samples were converted to 10% (w/v) suspensions in PBS (0.01 M, pH 7.2–7.4) immediately following the sampling. Total RNA was extracted from 100 ul suspension of each sample by using TRIZOL reagent (Invitrogen, USA) in accordance with the manufacturer's protocol. The viral RNA was finally dissolved in 20 μl RNase-free water. The primers used for HEV sequence amplification in this study were those previously described in reference [7]. The PCR products were analyzed in a 1.5% agarose gel. The expected DNA band specific for the HEV was excised from the gel, purified with the AxyPrep DNA Gel Extraction kit (Axygen, USA) and cloned into pMD T-Vector (TaKaRa, Japan). Both strands of the inserted DNA amplicons were sequenced in a DNA analyzer (Applied Biosystems 3730 DNA Analyzer; Invitrogen, USA). Standard precautions were used for all procedures to reduce the possibility of sample contamination and RNA degradation.

The detection results indicated one specimen from a seven months old infant was positive for HEV RNA. Sequence analysis based on the partial ORF2 sequence suggested that this HEV strain belonged to genotype 4. Then we determined the full-length nucleotide sequence of ORF2 of this strain using 3 sets of nested primers designed according the Chinese genotype 4 HEV isolates: EU366959, EF570133, and AB197674. The full ORF2 sequence was submitted to GenBank with the accession no.: FJ373295, and named Sh-hu-et1. The ORF2 of this isolate contain 2025 nt, and encodes a potential 674 aa polypeptide.

**Figure 1:**
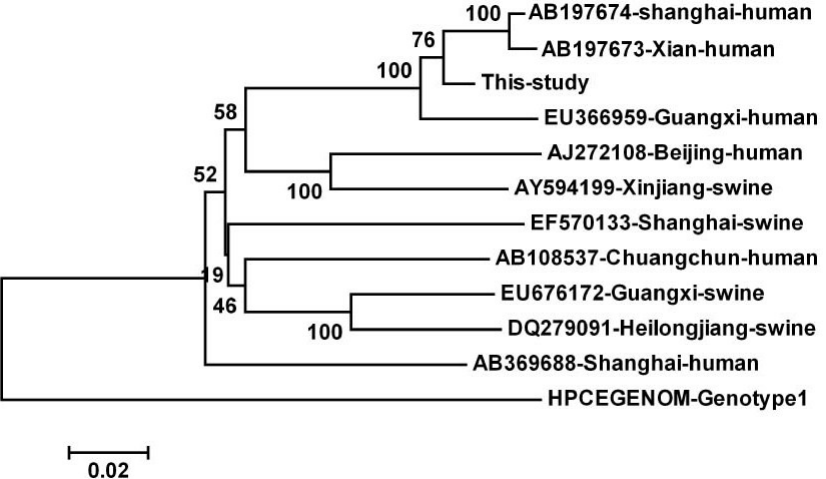
**Phylogenetic analysis based on the complete ORF2 sequence of the isolate in this study and other 10 genotype 4 HEV isolates in China, using the neighbor-joining method and evaluated using the interior branch test method with Mega 4 software**. Percent bootstrap support is indicated at each node. The scale bar represents nucleotide substitutions per base. GenBank accession no. and origin are indicated. A genotype 1 HEV strain was included as outgroup.

**Figure 2:**
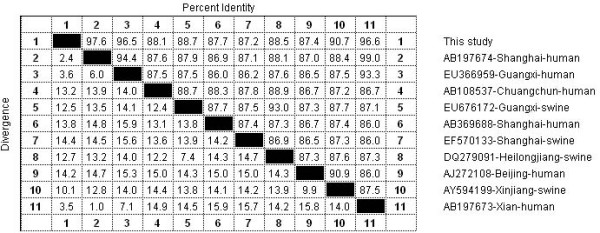
**Sequence analysis based on the complete ORF2 sequence of the isolate in this study and other 10 genotype 4 HEV isolates in China, using DNAstar software package**.

In order to investigate the evolutionary relationship of the HEV isolate in this study with other Chinese genotype 4 isolates, sequences were aligned using ClustaX v1.8 or MegAlign program in the DNASTAR software package. Phylogenetic tree was constructed using the Mega 4 software . Ten Chinese genotype 4 HEV strains with complete genome available in GenBank (including both swine and human original isolates) were used as references in the analysis; their GenBank accession numbers and source of origin are showed in Fig. 1. Sequence analysis (Fig. 2) based on full OFR2 sequences indicated the isolate in the present study shared 87.2–97.6% identities with the other Chinese genotype 4 HEV isolates referenced here, and the highest sequence identity (97.6%) with another Shanghai HEV strain (AB197674) isolated from a Japanese patient who was reportedly infected during traveling in Shanghai of China [8]. Phylogenetic analysis indicated that the isolate determined in this study closely clustered with other 3 Chinese genotype 4 HEV strains from human (Fig. 1), forming a subgroup. Interestingly, though the 4 HEV strains in this subgroup come from three geologically far regions: Shanghai (eastern China), Xi'an (western China) and Guangxing (southern China), they shared more than 90% nucleotide homologue with each other, suggesting they may come from a common ancestor isolate. Recent reports suggested that genotype 4 HEV isolates that prevalent in Shanghai humans showed phylogenetically close relationship with those swine genotype 4 HEV isolates commonly prevalent in Shanghai swine populations [9,10], suggesting that zoonotic transmission of genotype 4 HEV between human and swine populations was involved in this area. However, the isolate, Sh-hu-et1, in the current study and the other two previous Shanghai human isolates (AB197674, and AB369688) were phylogenetically far from the swine genotype 4 HEV isolate (EF570133) (Fig. 1) which was reported to be commonly prevalent in Shanghai swine groups [11], suggesting that some HEV strains that prevalent in Shanghai humans may not come from swine and didn't participate in zoonotic transmission. Further investigating research in swine groups should be aimed to determined the whether this genotype 4 HEV strain is prevalent in Shanghai swine or not.

HEV paly an important role in acute sporadic and epidemic viral hepatitis in Asia and Africa. Although HEV infection is frequent in young adults aged 15–20 years, it is uncommon in children and usually asymptomatic and anicteric [12]. Many reports indicated that anti-HEV IgG could be detected in serum of children [13], but few cases showed HEV RNA positive. In the present study, we detected HEV RNA in the fecal sample from a seven months old infant and this infant showed no clinical symptom of hepatitis, which suggested that infants may be sub-clinically infected by HEV.

## Conclusion

In conclusion, our study showed that a genotype 4 HEV strain infected a seven months old infant with no symptom of hepatitis in Shanghai Children's hospital. Sequence analysis based on the full ORF2 sequence indicated that this HEV strain shared the highest sequence identity with another human HEV strain isolated from a Japanese patient who was infected by genotype 4 HEV during traveling in Shanghai. Phylogenetic analysis showed that this genotype 4 HEV was phylogenetically far from the genotype 4 HEV strain that was commonly prevalent in Shanghai swine group, suggesting that this strain may not come from swine group and not involved in zoonotic transmission in this area.

## Competing interests

The authors declare that they have no competing interests.

## Authors' contributions

All authors participated in the planning of the project. XGH was the leader of the project. WZ, SXY, QS, and TLS collected all samples and performed the RT-PCR experiments for HEV RNA. WZ and SXY did the phylogenetic analysis. All authors read and approved the final manuscript.
